# Supplementary material for: Identification of photoperiod-induced specific miRNAs in the adrenal glands of Sunite sheep (Ovis aries)
Source: Front Vet Sci. 2022 Jul 22;9:888207. doi: 10.3389/fvets.2022.888207 (PMC9354845; doi:10.3389/fvets.2022.888207)
Supplement: Supplementary file 1 [file Data_Sheet_1.ZIP › Supplementary file/miRDeep2 file.zip!/miRDeep2 file/Know_Structure/SPLP42A3/oar-mir-134.pdf]

miRBase precursor : oar-mir-134
Total read count : 112
oar-miR-134-5p read count : 92
oar-miR-134-3p read count : 20
remaining reads : 0

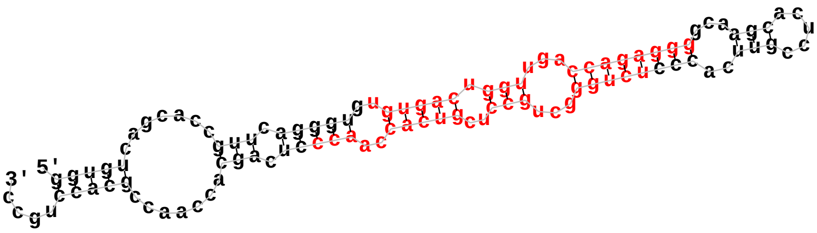

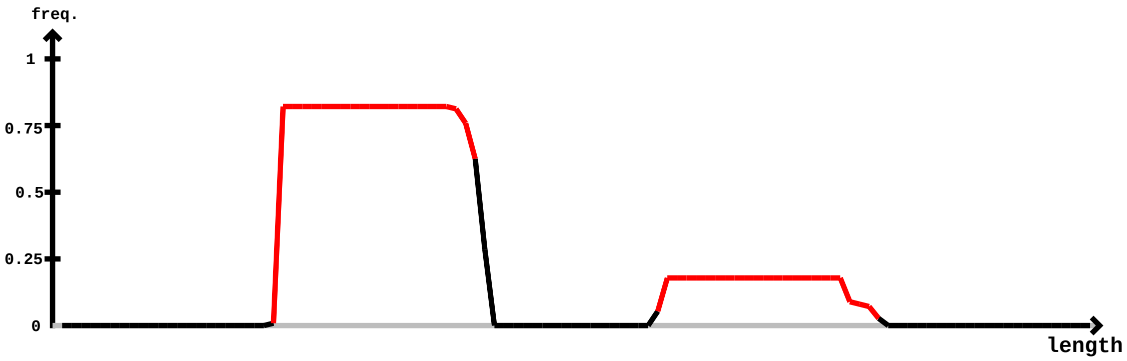

oar-miR-134-3p

oar-miR-134-5p

```
5'- ggugucagcaccguucagggugugugacugguugaccagagggggcaagcacuccguucacccucugggcugccucgucaccaacccucagcaccaaccgcaccugcc   -3'   exp
    (((((.......(((.(((((..(((((.(((...((((((((...(((.....)))..))))))))...)))..)))))..))))).))).......)))))....   reads   mm   sample
    .....................gugugacugguugaccaga...................................................................   1       0    seq
    ......................ugugacugguugaccagag..................................................................   6       0    seq
    ......................ugugacugguugaccagagg.................................................................   15      0    seq
    ......................ugugacugguugaccagaggg................................................................   38      0    seq
    ......................ugugacugguugaccagagggg...............................................................   32      0    seq
    .............................................................cucugggcugccucgucacc..........................   4       0    seq
    .............................................................cucugggcugccucgucacca.........................   1       0    seq
    .............................................................cucugggcugccucgucaccaa........................   1       0    seq
    ..............................................................ucugggcugccucgucacc..........................   6       0    seq
    ..............................................................ucugggcugccucgucaccaac.......................   5       0    seq
    ..............................................................ucugggcugccucgucaccaacc......................   3       0    seq
```
